# Compromised Brain Activity With Age During a Game-Like Dynamic Balance Task: Single- vs. Dual-Task Performance

**DOI:** 10.3389/fnagi.2021.657308

**Published:** 2021-07-05

**Authors:** Veerle de Rond, Diego Orcioli-Silva, Bauke Wybren Dijkstra, Jean-Jacques Orban de Xivry, Annette Pantall, Alice Nieuwboer

**Affiliations:** ^1^Neuromotor Rehabilitation Research Group, Department of Rehabilitation Sciences, KU Leuven, Leuven, Belgium; ^2^Posture and Gait Studies Laboratory (LEPLO), Institute of Biosciences, São Paulo State University (UNESP), Rio Claro, Brazil; ^3^Motor Control and Neuroplasticity Research Group, Department of Movement Sciences, KU Leuven, Leuven, Belgium; ^4^Leuven Brain Institute, Leuven, Belgium; ^5^Clinical Ageing Research Unit, Institute of Neuroscience, Newcastle University Institute of Ageing, Newcastle upon Tyne, United Kingdom

**Keywords:** fNIRS, aging, dual-task, cognitive load, postural control, weight-shifting

## Abstract

**Background:** Postural control and cognition are affected by aging. We investigated whether cognitive distraction influenced neural activity differently in young and older adults during a game-like mediolateral weight-shifting task with a personalized task load.

**Methods:** Seventeen healthy young and 17 older adults performed a balance game, involving hitting virtual wasps, serial subtractions and a combination of both (dual-task). A motion analysis system estimated each subject's center of mass position. Cortical activity in five regions was assessed by measuring oxygenated hemoglobin (HbO_2_) with a functional Near-Infrared Spectroscopy system.

**Results:** When adding cognitive load to the game, weight-shifting speed decreased irrespective of age, but older adults reduced the wasp-hits more than young adults. Accompanying these changes, older adults decreased HbO_2_ in the left pre-frontal cortex (PFC) and frontal eye fields (FEF) compared to single-tasking, a finding not seen in young adults. Additionally, lower HbO_2_ levels were found during dual-tasking compared to the summed activation of the two single tasks in all regions except for the right PFC. These relative reductions were specific for the older age group in the left premotor cortex (PMC), the right supplementary motor area (SMA), and the left FEF.

**Conclusion:** Older adults showed more compromised neural activity than young adults when adding a distraction to a challenging balance game. We interpret these changes as competitive downgrading of neural activity underpinning the age-related deterioration of game performance during dual-tasking. Future work needs to ascertain if older adults can train their neural flexibility to withstand balance challenges during daily life activities.

## Introduction

Older adults (OA) show deficits in postural control (Park et al., 2016) and dynamic weight-shifting (De Vries et al., 2014), which have been linked to an increase in fall risk (Delbaere et al., 2010). More specifically, disequilibrium in the mediolateral direction is a prominent predictor of unstable gait and falls (Cofré Lizama et al., 2015). The risk of falling also increases with cognitive distraction and dual-tasking (Muhaidat et al., 2014; Li et al., 2018), possibly because a significant attentional demand is required for postural control with age (Woollacott and Shumway-Cook, 2002). To counteract fall risk and its devastating consequences on mobility and physical activity, technology-based fall prevention programs are now widely advocated (Del Din et al., 2020). Exergames and interactive virtual reality (VR) platforms are used in the home as well as in rehabilitation settings, requiring high attentional engagement. Understanding brain activity changes during performance of a challenging balance game with and without cognitive distraction is a first step in grasping the neural impact of these task loads on OA.

Functional near-infrared spectroscopy (fNIRS) is an optical neuroimaging technique, which measures oxygenated (HbO_2_) and deoxygenated hemoglobin (HHb). Cortical activity is estimated based on the principle of neuro-vascular coupling (Bunce et al., 2006), whereby HbO_2_ levels indicate neural activation and HHb levels deactivation (Cui et al., 2010). fNIRS can measure brain activity in both young adults (YA) and OA while performing postural tasks in the upright position. Most studies have investigated the pre-frontal cortex (PFC) only, comparing single- (ST) and dual-task (DT) conditions during gait (Holtzer et al., 2011; Beurskens et al., 2014; Fraser et al., 2016; Takeuchi et al., 2016; Mirelman et al., 2017) and during balance tasks (Marusic et al., 2019). Two recent fNIRS studies also included the premotor cortex (PMC), supplementary motor area (SMA) and primary motor cortex (M1) during gait (Stuart et al., 2019a) and dynamic balance (Rosso et al., 2017). Overall, two studies observed no effect of either age or task on PFC-HbO_2_ levels (Takeuchi et al., 2016; Marusic et al., 2019). One study found a decrease in PFC during DT compared to ST in OA (Beurskens et al., 2014), whereas other studies found an increase, irrespective of age in PFC (Holtzer et al., 2011; Fraser et al., 2016; Mirelman et al., 2017), PMC and M1 (Stuart et al., 2019a). Two other groups showed a higher increase from ST to DT in YA compared to OA in PFC (Holtzer et al., 2011) and SMA (Stuart et al., 2019a), while one investigation observed a higher increase of PFC in OA compared to YA (Rosso et al., 2017).

The inconsistency of the previous findings may be explained by the diverse motor and cognitive tasks investigated (Woollacott and Shumway-Cook, 2002) and the lack of personalization of tasks. The majority of fNIRS-studies investigated the PFC only, precluding a more comprehensive understanding of cortical adaptations. In addition, motor and cognitive STs were not investigated separately to gain insight in the neural mechanisms of DT activation relative to that of the summed activations during separate STs. Leone et al. (2017) proposed that decreased brain activity during DT relative to the summed ST-activations, a so-called under-additive activation, could mean more efficient activation if behavioral DT performance remains stable. Alternatively, in case of a decline in behavioral DT performance, they suggested that relative DT deactivation could signify an inability to recruit sufficient neural resources due to limited brain capacity. To date, only one fNIRS study has made this comparison, showing a DT-related reduction of HbO_2_ levels in both young and older adults during a postural control task (Rosso et al., 2017).

The primary aim of this study is to investigate the impact of cognitive load and aging on HbO_2_ levels in frontal, motor and sensory cortical regions during a virtual reality mediolateral (ML) weight-shifting task, shown previously to be a feasible balance game for OA (Willaert et al., 2020). In line with most previous findings (Holtzer et al., 2011; Fraser et al., 2016; Mirelman et al., 2017; Rosso et al., 2017; Stuart et al., 2019a), we expect an overall increase in HbO_2_ levels during DT compared to ST, and this most probably in the PFC. To understand the possible competitive neural resources of DT during this challenging balance game, we will also examine DT brain activity changes relative to the summed ST activation. HHb levels will be analyzed as secondary outcomes.

## Materials and Methods

### Participants

Seventeen YA were recruited from the psychology student cohort at Newcastle University (UK). Seventeen OA were recruited through advertisements in the local Elders Council newsletter and various research support groups around the Newcastle region. All participants received an information letter and signed an informed consent prior to the experiment. Inclusion criteria included: (1) between 18 and 30 years for YA; (2) between 65 and 85 years for OA and (3) able to stand upright independently for at least 5 min. We used the Montreal Cognitive Assessment (MoCA) (Nasreddine et al., 2005) to evaluate cognition. Participants were excluded if they had cognitive impairment (MoCA <26), or a self-reported history of comorbidity, including neurological disorders, balance impairments (i.e., vestibular disorders), abnormal or uncorrected vision, acute pain, chronic musculoskeletal conditions, cardiovascular or respiratory conditions, or diabetes. After screening, participants underwent a cognitive test battery: (1) the Flanker test and (2) the Set-Shifting test, assessing inhibition and set-shifting (Kramer et al., 2020); and (3) the Benton Judgement of Line Orientation test (JLO) for visuospatial function (Benton, 1994). Participants also performed the MiniBEST (Franchignoni et al., 2010) to assess balance and completed a questionnaire on the concern of falling, the Falls efficacy Scale-International (FES-I) (Yardley et al., 2005) as well as a sarcopenia questionnaire, the SARC-F (Malmstrom et al., 2016). This study was approved by the Newcastle University Ethics Committee (6761/2018).

### Experimental Procedure and Tasks

#### Procedure

This study consisted of a cross-sectional comparison between age groups during one test session at the movement laboratory of Newcastle University. YA and OA performed a weight-shifting task as part of an exergame, the so-called wasp game (Willaert et al., 2020), a serial subtraction task and a combination of both (DT). Cortical activity was estimated with an fNIRS-system. The order of task conditions was counterbalanced across groups. Before starting the experiment, participants performed a 1-min practice trial in the wasp game ST (ST_wg_) and DT task conditions without the fNIRS-system for task-familiarization.

#### Weight-Shifting Task – Wasp Game

The wasp game was designed to assess weight-shifting ability in a game-like environment, and was developed at the department of Rehabilitation Science of KU Leuven, Belgium (Willaert et al., 2020) in collaboration with Motek Medical BV (Amsterdam, The Netherlands), using D-Flow software (Motek Medical BV, Amsterdam, The Netherlands). The wasp game is a personalized postural control task, requiring weight-shifts to obliterate “wasps” in a virtual reality environment. Functional limits of stability were assessed initially for each participant by moving the center of mass (CoM) as far as possible over its base of support in eight directions in one smooth movement (de Vries et al., 2018). These stability limits were used for personalized scaling of the wasp game. Motion was captured with a 3D-VICON-system. Eight reflective markers were placed bilaterally on the acromia, lateral epicondyles, posterior superior iliac spines (PSIS) and lateral malleoli. Six infrared 3D motion capture cameras recorded marker positions in Nexus software (Vicon, Oxford Metrics, UK) at a frequency of 100 Hz. Marker positions were transferred into D-Flow software (version 3.26) at a frequency of 300 Hz to calculate the CoM location in near real-time.

**Figure 1:**
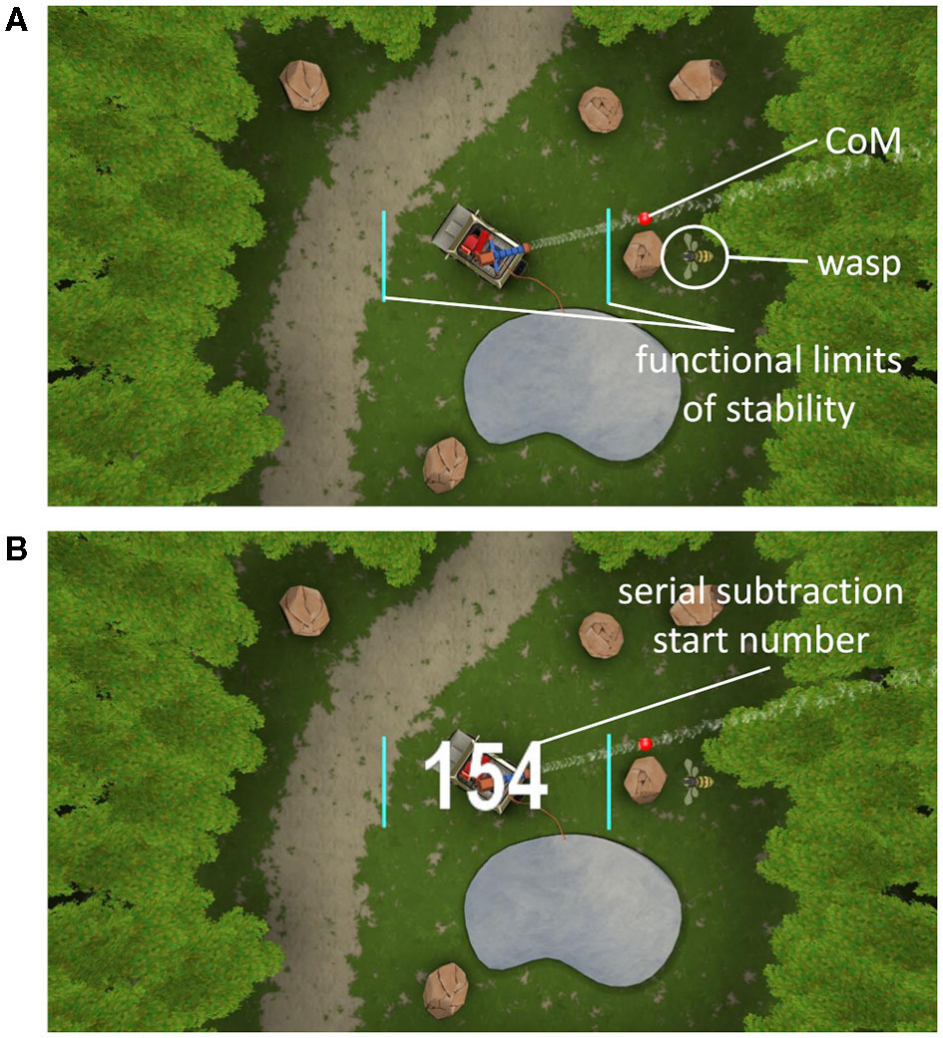
Weight-shifting wasp game **(A)**. The turquoise lines indicate 80% of the individual's lateral limits of stability. The red dot displays the center of mass (CoM). By moving the CoM laterally beyond the turquoise lines, the water jet was activated to hit the wasps that appeared on the left or right side of the screen alternately. Start screen of the serial subtraction single- and dual-task **(B)**. The white number represents the starting number, which was visible during the first 1.5 s only.

During the wasp game, participants' CoM was displayed by a red ball in the middle of an area “infested by wasps” (see Figure 1A). By moving the CoM beyond 80% of individual limits of stability, a water jet was activated to hit the wasps. One wasp was visible at a time, after which a new wasp appeared. Participants were instructed to hit as many wasps as possible. As disequilibrium in the ML direction is particularly predictive of falls (Cofré Lizama et al., 2015), only ML weight-shifts were performed. Foot placement was standardized by using tape markers on the floor, indicating a heel separation of 11% body height with an angle of 14° between the feet (Cofré Lizama et al., 2014). The wasp game was displayed on a projector screen located ~3 meters in front of the participant. Participants were instructed to cross their arms over their chest and perform weight-shifting movements from the ankle joints, while being discouraged to use hip movements and shoulder turns. The speed and accuracy (CoM trajectory error) of the ML shifts as well as the number of wasps hit were used as outcome measures.

#### Cognitive Task – Serial Subtraction

Similar to Marusic et al. (2019) and Mirelman et al. (2017), we used a serial subtraction task as the secondary cognitive task, as it allowed unobtrusive integration within the wasp game environment and precluded speech production so as not to interfere with the fNIRS signal. This kind of working memory task has shown to elicit a dual-task cost (Patel et al., 2014) and a change in PFC activation (Pelicioni et al., 2019) during walking. First, participants performed serial subtractions as ST (ST_ss_) during quiet stance, by watching a video of the wasp game, creating the same visual background as in the DT. Next, the serial subtractions and the weight-shifting game were performed simultaneously. At the beginning of each serial subtraction task, a randomly selected starting number between 150 and 200 for YA and between 50 and 100 for OA appeared on screen (see Figure 1B) to counter learning effects. Task onset was visualized by the ball that represented participants' CoM changing color from red to white and vice versa within a random interval of 2–5 s. YA were asked to subtract sevens and OA threes from the starting number. This procedure was applied to equalize cognitive load, optimize comparison of the fNIRS signals and achieve a similar impact of the DT, regardless of age. Participants were requested not to verbalize their calculations and to only say the final number at the end of each trial. The correct final number was calculated within the software, allowing for the assessment of the calculated final number being “correct” or “incorrect.” As such, the scores on the cognitive task ranged from 0 to 5 within both ST_ss_ and DT with 1 point to be earned during each trial.

#### Study Design

**Figure 2:**
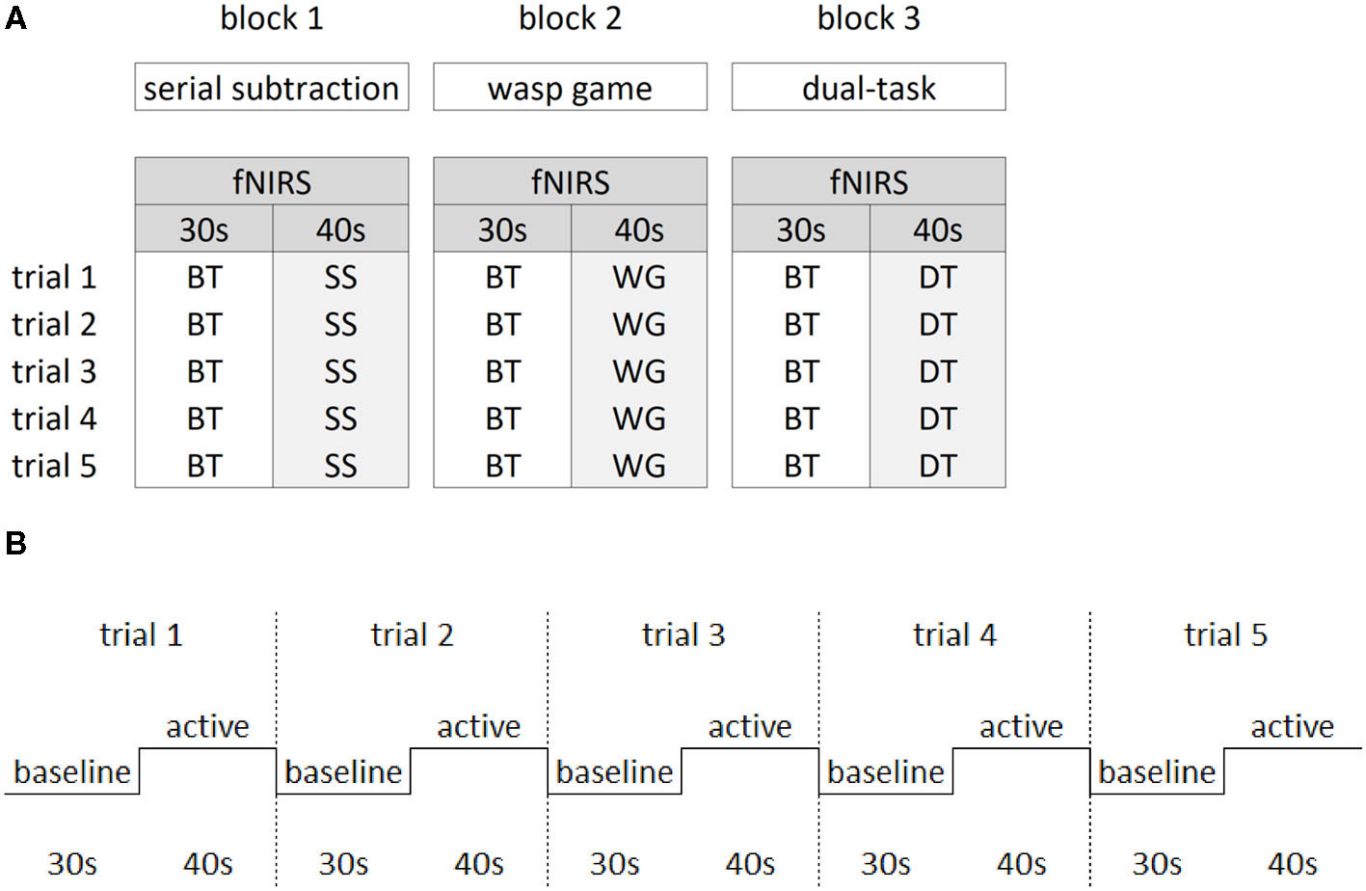
Study design. The block design **(A)** consisted of three blocks of the main manipulations of interest. Within each block, the baseline task (BT) was alternated with either the serial subtraction task (SS), the wasp game (WG) and both SS and WG combined in the dual-task (DT). Per block, five trials were collected for the active and baseline tasks. The latter consisted of standing while watching the screen with a video of the wasp task only, in which visual input from the wasps flying was similar to the active tasks. The repeated block design over five trials **(B)** consisted of 30 s of baseline task alternated with 40 s of active task.

Figure 2A shows that a block design was implemented varying baseline with active task performance to allow calculation of task-related fNIRS changes. During baseline, participants were asked to stand as still as possible while watching a video of the wasp game to control for the visual input of the VR interface. Participants completed three blocks of 5 min and 50 s. In each block, five baseline trials (30 s) were alternated with five active trials (40 s) (see Figure 2B) in which each ST (ST_wg_ or ST_ss_) and the DT were performed. All three blocks were randomized between participants. Rest periods were offered in between blocks, followed by 1-min of quiet stance before starting the next block to ensure stabilization of cerebral blood flow.

#### fNIRS Assessment

**Figure 3:**
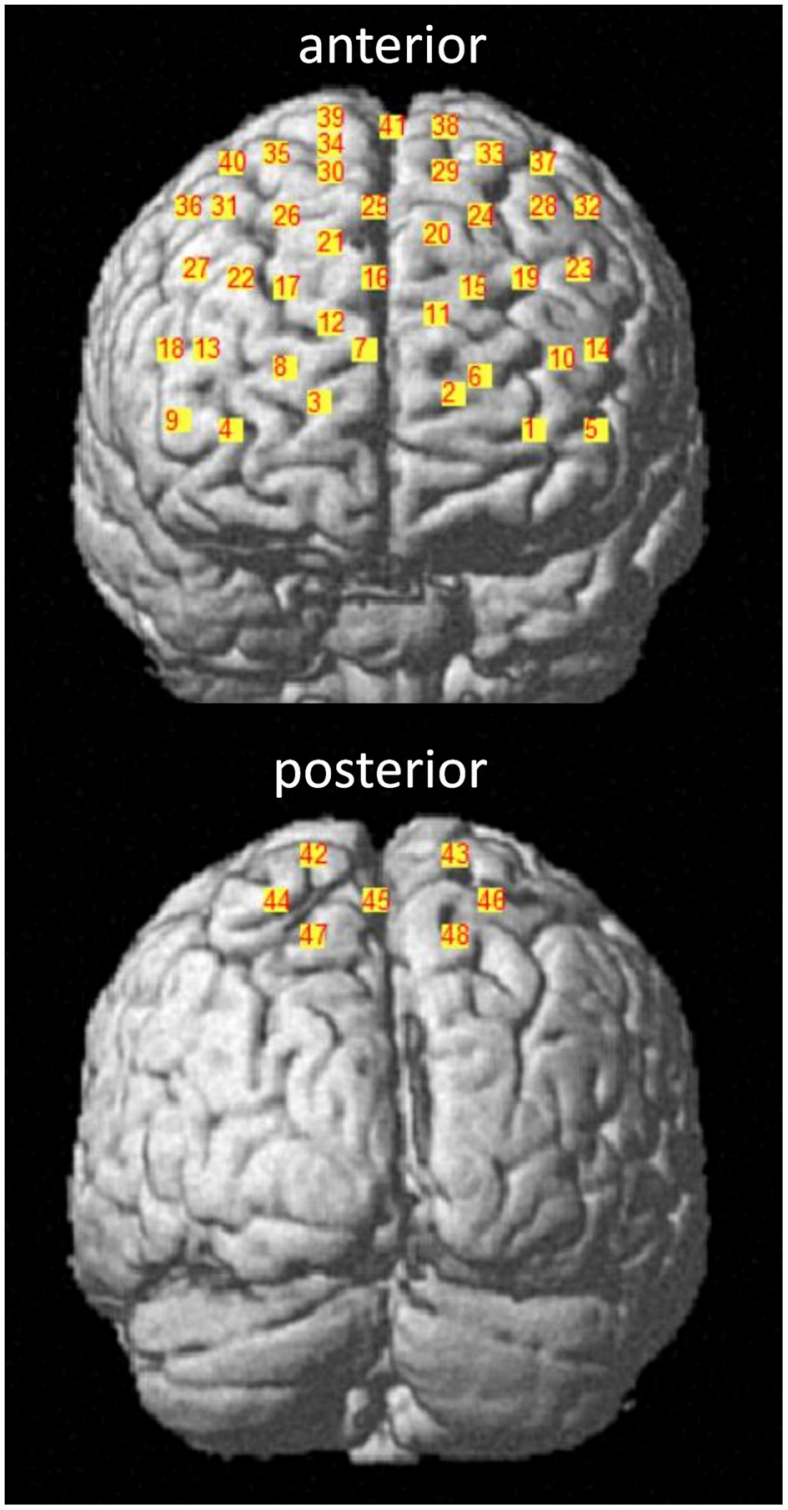
fNIRS channel locations. Channels are displayed in the anterior and posterior plane with corresponding channel numbers for a representative participant. The 10–20 EEG system was used as a reference. Channel 41 corresponds to the Cz landmark.

For the assessment of the hemodynamic brain response, which has been shown to correlate with neural activity (Fabiani et al., 2013), a tethered fNIRS-system (LABNIRS, Shimadzu, Kyoto, Japan) was used. The fNIRS system used continuous wave laser diodes with wavelengths of 780, 805, and 830 nm to measure the HbO_2_ and HHb at a sampling frequency of 23.8 Hz. Further details of this system, including HbO_2_ and HHb conversion coefficients, are published in Stuart et al. (2019a). The nasion, inion, and Cz anatomical landmarks for each participant were identified prior to placement of the fNIRS cap. Thirty-two optodes (16 sources, 16 receivers) were attached to the cap with a source-detector separation of ~3 cm, generating 48 channels as shown in Figure 3. To control for different head sizes, a 3D-digitizer (FASTRAK, Polhemus, VT, USA) was used to record the co-ordinates of the nasion, inion, Cz, left, and right pre-auricular points, as well as the optode locations. In addition, heart rate and oxygen saturation were monitored throughout the experiment with a wireless oximeter (Onyx II 6590, Nonin Medical Inc, MN, USA) clipped to the index finger and processed with Nonin software (OEM Evaluation Kit Program Files).

### Data Processing

#### Behavioral Data

The CoM data were analyzed within Matlab 2018b (Mathworks, MA, USA). First, marker data were low-pass filtered (Butterworth, cut-off = 6Hz, order = 4), after which the CoM position was calculated based on Winter (2009). In short, CoM positions of the trunk and leg segments were first calculated separately and then combined in a total CoM position. Supplementary Figure 1 shows the weight-shifting trajectories during ST_wg_ for a representative young and older adult in the ML direction. A weight-shift movement was defined as the movement from the last peak on the right to first peak on the left and vice versa. Here, the peaks represent the moment that weight-shifting speed is equal to zero. As such, the first weight-shift started after the first wasp was hit. A visualization of the CoM outcome parameters is displayed in Supplementary Figure 2. Speed and accuracy (weight-shifting error) of the ML shifts were computed for each shift separately and then averaged across shifts per trial. Next to the absolute outcome measures, the %DT-cost was calculated as the difference between ST_wg_ and DT performance relative to ST_wg_ performance [(ST_wg_-DT)/ST_wg_ * 100] (Friedman et al., 1982) for each participant individually, when at least a significant main effect of task was present. DT-cost of the serial subtraction task (number of correct scores) was calculated as the absolute difference between DT and ST_ss_ performance (ST_ss_-DT), as dividing this difference by zero was not possible.

#### fNIRS Data

We selected ROIs based on the gait literature due to the lack of studies in the balance domain. Previous work on animals suggested that motor planning areas, like the supplementary motor area (SMA; Brodmann area 6, medial), and the premotor cortex (PMC; Brodmann area 6, lateral) are involved in maintaining stability during gait (Takakusaki, 2017). In addition, both SMA and PMC have been shown to be involved in postural control (Wittenberg et al., 2017). The SMA also contributes to motor adaptation during imagined locomotion (la Fougère et al., 2010). The PMC is highly involved in the planning of visually guided gait (Wagner et al., 2014; Nakajima et al., 2019) and in DT gait in YA as captured with fNIRS (Lu et al., 2015). The somatosensory cortex (SSC; Brodmann areas 3, 5, and 7) has been found to be important for sensorimotor processing during walking in a virtual environment when assessed with EEG (Wagner et al., 2014). Furthermore, the pre-frontal cortex (PFC; Brodmann areas 9, 10, 11, 45, 46, and 47) has consistently been shown to be a central hub in executive function network, related to fall risk in patients with Parkinson's disease (Rosenberg-Katz et al., 2015) and active during the performance of a postural DT (Li et al., 2018). Finally, the frontal eye fields (FEF; Brodmann area 8) are active during control of eye-movements and visual cognition (Vernet et al., 2014), and were included due to the visually-driven nature of the VR game.

We applied recently published recommendations (Vitorio et al., 2017; Menant et al., 2020) to record fNIRS signals, except when precluded by technical specifications of the system (e.g., no short-separation channels were integrated). Three-dimensional co-ordinates were evaluated within the spatial registration routine (stand-alone NIRS) available in NIRS-SPM and implemented in Matlab 2010 (Mathworks, MA, USA). After evaluation, spatial locations of each channel were downloaded as MNI coordinates and Brodmann areas (Talairach daemon). A channel was only included if the Brodmann areas covered one ROI for at least 80% (Noah et al., 2015). As Brodmann areas do not distinguish between the SMA and PMC, the MNI co-ordinates for these channels were checked in the human motor area template (Mayka et al., 2006) using the MRIcron tool. For all ROIs, the left and right hemisphere were analyzed separately.

Pre-processing of fNIRS signals was performed for each block separately using the time series analysis routine in the freely available NIRS-SPM software (version 4, http://www.nitrc.org/projects/nirs_spm), and included the following steps:

1) Low-pass filtering (cut-off 0.15 Hz) based on canonical hemodynamic response function (Friston et al., 2000).
2) De-trending of the signal using a wavelet-minimum description length, based on a de-trending algorithm. The signal was broken down into global trends, hemodynamic signals, and uncorrelated noise components (Jang et al., 2009).
3) Baseline correction by setting the baseline to the initial time of the first trial. To ensure analyzing good quality signals only, signals with a poor connection as determined by the fNIRS Shimadzu system's automatic adjustment were excluded from further analysis (1.8% signal exclusion).
4) Signal quality was determined using a hybrid method. First, 20% of all signals obtained were visually inspected by two independent raters. Several criteria were used for signal selection. The most important one was based on rating the divergence between HbO_2_ and HHb signals, as previously used (Stuart et al., 2019b). This procedure had an overall inter-rater agreement of 88%. As the HbO_2_ and the HHb neural signals are expected to be negatively correlated (Cui et al., 2010), an anti-correlation analysis was performed, as this has been shown to improve signal quality in the absence of short-separation channels (Zhou et al., 2020). Signals with a positive correlation (*r* > 0) were excluded from further analysis. The agreement between observer-based exclusion and correlation-based exclusion was 80% for rater 1 and 82% for rater 2.
5) The last steps of signal processing performed on the final selection of fNIRS signals was done in Matlab 2018b (Mathworks, MA, USA). Channels were averaged per ROI and normalized by dividing the averaged signal by the maximum range across trials (Koenraadt et al., 2014), thereby calculating proportional HbO_2_ and HHb levels. This normalization process allows for between-group comparison. The first and last 5 s of each condition were excluded from the analysis to control for the temporal delay in the fNIRS signal. Signals obtained from the baseline condition (standing still while watching the wasp-game video) were subtracted from that of the active conditions, creating relative normalized HbO_2_ and HHb levels (expressed as ratio values).

HbO_2_ was used as the primary outcome measure as earlier study showed that it had a better contrast-to-noise ratio than HHb (Strangman et al., 2002). HHb outcomes were reported when significant group by task interactions were different from those found in HbO_2_ (i.e., HHb did not mirror HbO_2_ only). The HbO_2_ and HHb levels of the ST_wg_ and ST_ss_ were added together (ST_wg_+ST_ss_) in order to investigate brain activity changes during DT.

### Statistical Analysis

Demographic characteristics and behavioral performance during ST_wg_ and ST_ss_ were compared between age groups using independent samples *t*-tests or Mann-Whitney *U*-tests, depending on the normality of data distribution. To investigate the effect of a DT on both behavioral and cortical parameters, a linear mixed model approach was chosen to account for missing values (IBM SPSS Statistics 26) (Boisgontier and Cheval, 2016). Subject number was included as random factor, and group (YA vs. OA) and task (model 1: ST_wg_ vs. DT; model 2: ST_wg_+ST_ss_ vs. DT) as fixed factors, allowing to model for main effects of group, task, trial, and the group^*^task interactions. Heart rate and oxygen saturation were included as covariates for the fNIRS-analysis. Before running the models, assumptions of homoscedasticity, linearity, and normality of residuals were checked. As no significant trial effect was found, unlike in previous studies (Holtzer et al., 2019; Stuart et al., 2019a), data across trials were pooled for further analysis. Bonferroni correction was applied for all *post-hoc* testing of significant interaction effects. Finally, Pearson's correlations were calculated for each age group separately to investigate the relationship between participants' behavioral measures and neural outcomes of significant *post-hoc* tests only (12 possible combinations). As these correlations were exploratory in nature, no correction for multiple comparisons was applied. All *p*-values were set at 0.05 for significance.

## Results

### Baseline Between-Group Differences

Table 1 shows that age groups were comparable for global cognition. However, OA showed worse balance (MiniBEST scores and limits of stability; see for details Supplementary Figure 3) and executive function (Flanker and Set-Shifting test) than YA. In ST_wg_, OA hit less wasps than YA [*t*(32) = 3.930, *p* < 0.001] and shifted their bodyweight more slowly [*t*(32) = 4.235, *p* < 0.001]. However, weight-shifting error was not significantly larger for OA compared to YA [*t*(32) = −0.490, *p* = 0.314]. The two groups also had a similar performance on ST_ss_ (see Table 1).

**Table 1:** Demographics, cognitive test scores, wasp game ST, and serial subtraction ST performance for young and older adults.

	**Young adults**	**Older adults**	***p*-value**
**Demographics**			
Sex (m/f)	7/10	5/12	0.086^a^
Age (years)	20.0 (19, 21)	77.0 (69.5, 79.0)	**<0.001**
Height (cm)	1.74 ± 0.13	1.65 ± 0.08	**0.013**
Weight (kg)	63.0 (59.5, 78.6)	64.4 (60.0, 71.4)	0.973
SARC-F (0–10)	0.0 (0.0, 1.5)	0.0 (0.0, 1.0)	0.919
FES-I (16–64)	18 (16.5, 23)	18 (16.5, 21.5)	0.734
MiniBEST (0–28)	27 (26.5, 27.5)	23 (21, 24.5)	**<0.001**
**Cognitive tests**			
MoCA (0–30)	27.0 (26.5, 28.0)	28.0 (27.0, 29.0)	0.114
Flanker (0–10)	9.3 ± 0.4	8.4 ± 0.3	**<0.001**
Set-Shifting (0–10)	9.0 (8.3, 9.3)	7.4 (5.7, 8.1)	**<0.001**
JLO (0–30)	26 (23.5, 28)	25 (18.5, 28)	0.357
**ST performance**			
#Correct answers - SS (0–5)	0.0 (0.0, 0.5)	0.0 (0.0, 1.0)	0.306
#Wasps hit - wasp game	14.56 ± 4.00	10.12 ± 3.08	**<0.001**
CoM speed - wasp game (m/s)	0.110 ± 0.034	0.067 ± 0.027	**<0.001**
CoM error - wasp game (m)	0.005 ± 0.002	0.006 ± 0.002	0.627

*Normally distributed data are displayed as mean ± SD, and not normally distributed data as median (first quartile, third quartile). ST, single-task; SARC-F, Sarcopenia Questionnaire; FES-I, Falls Efficacy Scale International; MiniBEST, Mini Balance Evaluation Systems Test; MoCa, Montreal Cognitive Assessment; JLO, Judgement of Line Orientation; SS, Serial Subtraction; CoM, center of mass*.

a *X^2^. Bold values indicate significant group differences (p < 0.05)*.

### Behavioral Results

#### Weight-Shifting in ST vs. DT

**Figure 4:**
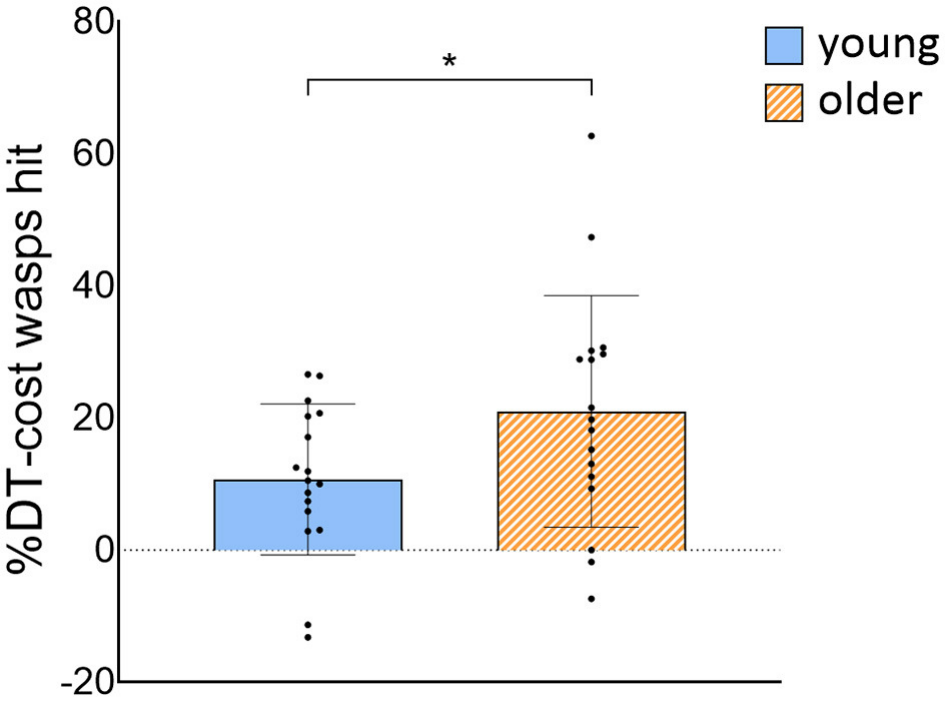
%DT-cost of the number of wasps hit for young and older adults. %DT-cost was calculated as the difference between DT and ST_wg_ performance as a percentage of ST_wg_ performance, and is displayed as mean ± SD. Individual data points represent %DT-cost for each participant. *Significant group difference (*p* < 0.05). ST_wg_, single-task wasp game; DT, dual-task.

OA hit less wasps [group effect: *F*_(1, 32)_ = 22.054, *p* < 0.001] and had slower weight-shifting speed [group effect: *F*_(1, 32)_ = 21,656, *p* < 0.001] than YA, irrespective of task load. Also, less wasps were hit [task effect: *F*_(1, 300)_ = 125.127, *p* < 0.001] and weight-shifting was slower [task effect: *F*_(1, 300)_ = 71.658, *p* < 0.001] in DT compared to ST_wg_. We did not find evidence that these decreases in wasps hit [interaction effect: *F*_(1, 300)_ = 2.037, *p* = 0.155] and in weight-shifting speed [interaction effect: *F*_(1, 300)_ = 0.236, *p* = 0.628] from ST_wg_ to DT differed across groups. Yet, when wasps hit were calculated as a ratio of the ST_wg_ (%DT-cost), OA showed a significantly higher %DT-cost than YA [*t*_(32)_ = −2.032, *p* = 0.025; see Figure 4]. The %DT-cost was not different between groups for weight-shifting speed. No group, task, or interaction effects were found for weight-shifting error (see Table 2).

**Table 2:** Weight-shifting outcomes for young and older adults during wasp game ST and DT.

	**Young adults^a^**		**Older adults^a^**		**Group main effect**	**Task main effect**	**Interaction effect**
	**ST_**wg**_**	**DT**	**ST_**wg**_**	**DT**			
#wasps hit	14.56 ± 4.00	12.84 ± 3.11	10.12 ± 3.08	7.98 ± 2.96	**<0.001**	**<0.001**	0.155
CoM speed (m/s)	0.110 ± 0.034	0.101 ± 0.029	0.067 ± 0.027	0.058 ± 0.020	**<0.001**	**<0.001**	0.628
CoM error (m)	0.005 ± 0.002	0.006 ± 0.002	0.006 ± 0.002	0.006 ± 0.002	0.780	0.051	0.639

*Data are displayed as mean ± SD. ST_wg_, single-task wasp game; DT, dual-task*.

a N = 17; Bold values indicate significant group main, task main and group*task interaction effects (p < 0.05).

#### Serial Subtraction in ST vs. DT

The number of correct final answers on the serial subtraction task showed a higher DT-cost with age (U = 212.5, *p* = 0.009). OA significantly decreased their cognitive performance during DT compared to ST_ss_ (3.7 vs. 15.7%, *Z* = 0.00, *p* = 0.012), whereas there was a non-significant improvement in YA (10.8 vs. 18.5%, *Z* = 15.5, *p* = 0.145).

### fNIRS Results

**Figure 5:**
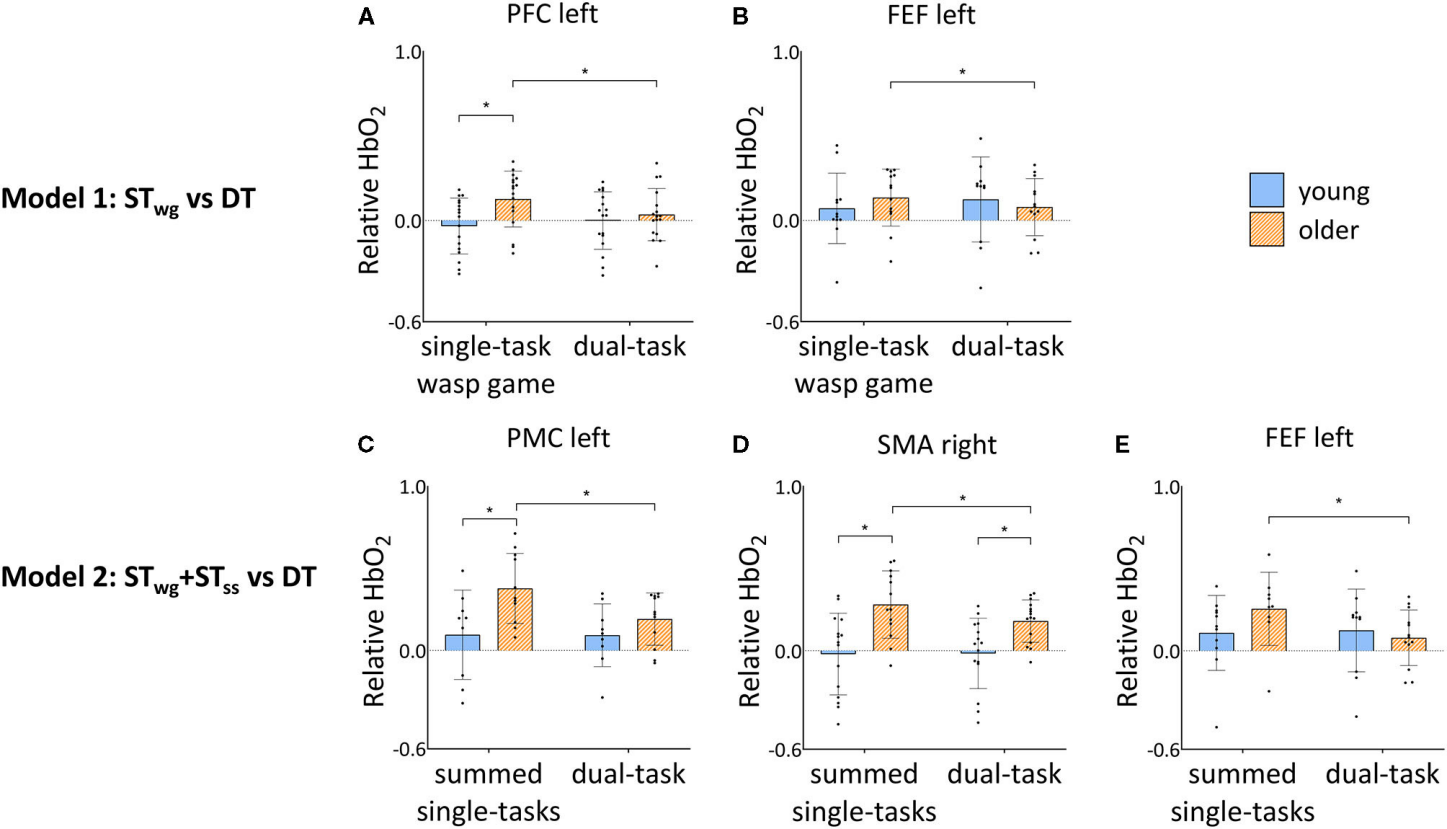
Group by task interaction effects in PFC left **(A)** and FEF left **(B)** HbO_2_ levels during ST_wg_ and DT, and FEF left **(C)**, PMC left **(D)** and SMA right **(E)** HbO_2_ levels during ST_wg_+ST_ss_ and DT in young and older adults. HbO_2_ levels are calculated relative to the baseline task and displayed as mean ± SD. Individual data points represent relative HbO_2_ levels for each participant. *Significant *post-hoc* effects (*p* < 0.05). ST_wg_, single-task wasp game; ST_ss_, single-task serial subtractions; DT, dual-task; PFC, pre-frontal cortex; FEF, frontal eye fields; PMC, premotor cortex; SMA, supplementary motor area.

Figure 5 shows the interactions on HbO_2_ levels found for both models (model 1: ST_wg_ vs. DT; model 2: ST_wg_+ST_ss_ vs. DT) and see also Supplementary Tables 1, 3 for the detailed results.

#### Cortical Activation During Weight-Shifting in ST vs. DT (Model 1)

Independent of ST_wg_ or DT, OA displayed higher HbO_2_ levels compared to YA in the right supplementary motor area (SMA) [group effect: *F*_(1, 35)_ = 12.63, *p* = 0.001] and right somatosensory cortex (SSC) [group effect: *F*_(1, 26)_ = 12.53, *p* = 0.002]. Group by task interaction effects were found in the left pre-frontal cortex (PFC) and frontal eye fields (FEF) only [left PFC: *F*_(1, 302)_ = 8.68, *p* = 0.003; left FEF: *F*_(1, 216)_ = 5.28, *p* = 0.022]. Results of *post-hoc* testing are illustrated in Figures 5A,B, showing that HbO_2_ levels were generally higher for OA compared to YA during ST_wg_ in the left PFC [*F*_(1, 50)_ = 10.58, *p* = 0.002], but not in the left FEF. In DT compared to ST_wg_, HbO_2_ levels were significantly decreased for OA in the left PFC [*F*_(1, 314)_ = 12.97, *p* < 0.001] and left FEF [*F*_(1, 233)_ = 4.18, *p* = 0.042], which was not seen in YA.

HHb levels, postulated to represent neural deactivation, mirrored the results found in the left PFC and right SMA HbO_2_ levels (see Supplementary Table 2). These results did additionally reveal a group by task interaction effect in the left SMA only [*F*_(1, 252)_ = 5.68, *p* = 0.018]. Negative HHb levels may indicate less deactivation during the active vs. the baseline tasks. *Post-hoc* testing showed that HHb levels were similar for both groups during ST_wg_ (see Supplementary Figure 4). During DT, HHb levels in YA became less negative [*F*_(1, 259)_ = 7.07, *p* = 0.008] suggesting more deactivation (or less activation) in the left SMA. Furthermore, HHb levels in OA were more negative during DT [*F*_(1, 49)_ = 9.81, *p* = 0.003] compared to YA, indicating that OA required less neural deactivation (or more activation) in this area.

#### The Sum of STs vs. DT Cortical Activations (Model 2)

In model 2, we investigate differences in DT cortical activity compared to the sum of the cortical activity recorded during the individual motor and cognitive STs (ST_wg_+ST_ss_). OA displayed overall higher HbO_2_ levels than YA in the left PFC [group effect; *F*_(1, 39)_ = 5.06, *p* = 0.030] and the SSC in both hemispheres [group effect; left: *F*_(1, 27)_ = 4.74, *p* = 0.034; right: *F*_(1, 25)_ = 9.50, *p* = 0.005]. Furthermore, the DT conditions generated lower HbO_2_ levels compared to ST_wg_+ST_ss_ in the left PFC [task effect: *F*_(1, 307)_ = 4.48, *p* = 0.035], the right FEF [task effect: *F*_(1, 288)_ = 12.62, *p* < 0.001], the right premotor cortex (PMC) [task effect: *F*_(1, 207)_ = 4.15, *p* = 0.043], the left SMA [task effect: *F*_(1, 239)_ = 6.35, *p* = 0.012], and the SSC in both hemispheres [left: *F*_(1, 220)_ = 12.86, *p* < 0.001; right: *F*_(1, 185)_ = 12.65, *p* < 0.001], see Supplementary Table 3.

Group by task interactions were found in the left FEF [*F*_(1, 194)_ = 17.07; *p* < 0.001], left PMC [*F*_(1, 190)_ = 10.01; *p* = 0.002], and right SMA [*F*_(1, 260)_ = 5.49; *p* = 0.020]. Results of the *post-hoc* testing are illustrated in Figures 5C–E showing that HbO_2_ levels were higher in OA compared to YA for ST_wg_+ST_ss_ in the left PMC [*F*_(1, 29)_ = 8.25; *p* = 0.008] and in the right SMA [ST_wg_+ST_ss_: *F*_(1, 42)_ = 17.79; *p* < 0.001; DT: *F*_(1, 46)_ = 7.01, *p* = 0.011]. In DT, HbO_2_ levels were significantly lower for OA in the left PMC [*F*_(1, 193)_ = 16.58; *p* < 0.001], the right SMA [*F*_(1, 255)_ = 11.02; *p* = 0.001], and the left FEF [*F*_(1, 201)_ = 30.12; *p* < 0.001] compared to ST_wg_+ST_ss_, which was not seen in YA.

Additionally, HHb levels revealed an interaction effect in the right FEF [*F*_(1, 285)_ = 5.27, *p* = 0.022]. *Post-hoc* testing showed that HHb levels were higher in DT compared to ST_wg_+ST_ss_ for YA [*F*_(1, 291)_ = 7.31, *p* = 0.007; see Supplementary Figure 5], whereas no difference was found in OA. See also Supplementary Table 4 for more details.

#### Correlations Between Weight-Shifting and Cortical Activity

Exploratory univariate correlation analyses were performed between the cortical regions which showed significant group by task interactions for HbO_2_ levels (left PFC, left FEF, and left SMA), and task performance in ST_wg_ and DT. During ST_wg_, HbO_2_ levels in the left PFC in OA were correlated with the number of wasps hit (*r* = 0.47, *p* = 0.029; see Supplementary Figure 6A). However, DT performance proved not correlated with HbO_2_ levels. HHb levels in the left SMA during ST_wg_ in YA were negatively correlated with weight-shifting speed (*r* = −0.44, *p* = 0.038; see Supplementary Figure 6B), a pattern that was not found in the OA and also ceased to be present during DT. No other correlations were revealed.

## Discussion

### Main Study Findings

**Figure 6:**
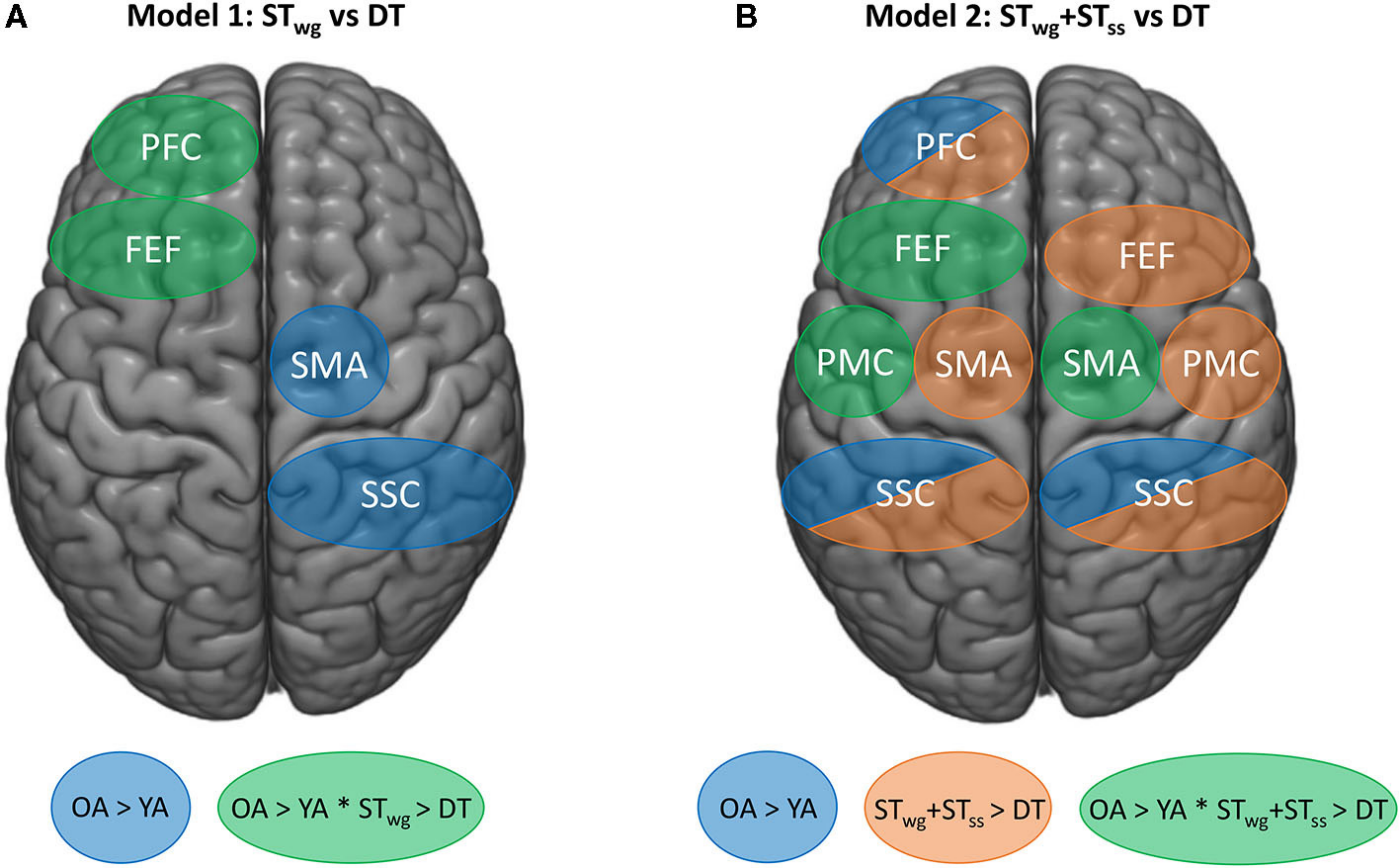
Schematic overview of the fNIRS findings on HbO_2_ levels for mixed model 1 (ST_wg_ vs DT) **(A)**, and mixed model 2 (ST_wg_+ST_ss_ vs. DT) **(B)**. Blue areas represent group effects (*p* < 0.05) with higher HbO_2_ levels in OA vs. YA. Task effects are displayed by orange areas (*p* < 0.05), representing lower HbO_2_ levels in the DT compared to ST_wg_+ST_ss_. Green areas represent interaction effects (*p* < 0.05) with higher HbO_2_ levels in OA vs. YA during ST_wg_ and ST_wg_+ST_ss_ and greater HbO_2_ level reductions in OA vs. YA during DT. ST_wg_, single-task wasp game; ST_ss_, single-task serial subtractions.

This is the first study that investigated the effects of combining a serial subtraction task with an individualized weight-shifting game on cortical activity as measured with fNIRS in YA and OA. We demonstrated that OA showed a diminished game performance as well as a reduced serial subtraction task execution compared to YA, when performing both tasks simultaneously. Figure 6 shows an overview of the main neural results found. With age, higher HbO_2_ levels were present in the left PFC during ST_wg_. In contrast to our hypothesis, in which we expected an increase in HbO_2_ levels during DT compared to ST_wg_, OA showed reduced HbO_2_ levels in the left PFC and FEF. In contrast, HbO_2_ levels in YA did not change. Interestingly, HbO_2_ levels were lower during the DT compared to ST_wg_+ST_ss_ in all the ROIs investigated, except for the right PFC. In the left PMC, right SMA and left FEF, these reduced HbO_2_ levels were age-specific, as the reductions were found in OA only. This age-related downgrading of activity may signify neural competition, occurring when two tasks rely on similar neural resources, and as such explain why adding cognitive load to a challenging balance game had a larger impact on OA vs. YA even when task load was adapted to the age group.

### Behavioral Results

ML weight-shifting performance deteriorated with age as OA hit less wasps and had slower weight-shifting speed than YA in ST_wg_, even though the game was individualized to the stability limits of each subject. Stability limits were smaller for the OA than the YA, suggesting that the former group was using a more cautious strategy during weight-shifting in keeping with their reduced postural control and lower MiniBEST scores at baseline. In contrast to previous studies (Cofré Lizama et al., 2014; De Vries et al., 2014), no between-group difference in weight-shifting error (accuracy) was apparent. OA may have slowed down their performance in order to maintain an adequate level of accuracy during the wasp game.

When additionally performing serial subtractions, weight-shifting speed and wasp hits worsened in both groups, but more so in the OA for the wasp scores only. It is possible that, in line with the attentional capacity model (Leone et al., 2017), the volume of attention needed for each task was exceeded, leading to deteriorated task performance (see subsequent sections Cortical Activation During Weight-Shifting in ST vs. DT and Summed ST vs. DT cortical activations). Previous balance studies reported no group by task interactions while OA and YA combined tandem stance with a serial subtraction task (Marusic et al., 2019) or while standing on a dynamic balance platform with a choice reaction time task (Rosso et al., 2017). By comparison, the present weight-shifting paradigm combined with wasp-hitting was probably more challenging and therefore showed a group difference in %DT-cost.

ST_ss_ outcomes were similar between groups, possibly explained by adjusting the difficulty based on age at the group level. Controlling for secondary task load may have avoided over- or underestimation of dual-task interference in OA or YA, respectively, offering a better baseline for comparing the hemodynamic responses between groups. A dichotomous score (“correct” or “incorrect) was used to score performance, as speech was precluded during fNIRS data collection. Therefore, more subtle between-group differences may have been missed. During the DT, OA displayed less correct final responses, whereas YA showed no difference. This finding is in contrast to the findings of Marusic et al. (2019), who reported that YA improved their performance on a serial subtraction task during DT without impact on balance. Although Marusic et al. (2019) used a similar cognitive task, the postural task (tandem stance) was much easier than our wasp game, which included both visual, attentional and motor components. The inherent task difficulty of the wasp game might thus explain the differences in DT performance found. Also, in the present study, it appeared that OA lost proficiency in both task domains, whereas YA became less efficient in weight-shifting, while maintaining cognitive performance.

### fNIRS Results

#### Cortical Activation During Weight-Shifting in ST vs. DT

The fNIRS outcomes during the ST_wg_ revealed that OA had higher HbO_2_ levels in the left PFC than YA. Similar results were found previously in the left dorsolateral PFC when OA were standing on a dynamic balance platform (Rosso et al., 2017). Our exploratory correlation analysis suggested that higher PFC-HbO_2_ values were associated with more wasps hit during the game. As such, it is possible that this PFC activity increase reflected compensatory neural recruitment for the age-related weight-shifting decline (Heuninckx et al., 2008) and loss of executive function (Li et al., 2018). As no Bonferroni corrections were applied to this correlation analysis, this interpretation should be viewed with caution.

Figure 6A provides a schematic overview of the most important fNIRS findings. Contrary to our primary hypothesis, it reveals that when performing the wasp game in DT, HbO_2_ levels in the left PFC and FEF reduced (green regions) compared to ST_wg_ in OA but not in YA (Rosso et al., 2017; Stuart et al., 2019a). We speculate that these greater HbO_2_ reductions in OA could reflect an overload-induced deactivation (Leone et al., 2017), resulting in a loss of executive control over the wasp game due to the distraction. More specifically, the reduced left FEF-HbO_2_ levels may also have contributed to the fall in wasps hit, as this area is involved in visual attention and visual working memory (Vernet et al., 2014). However, no brain-behavior correlations for the left FEF were revealed in either ST_wg_ or DT, precluding a firm interpretation of these results.

It could also be that the compensatory activity in the PFC during ST_wg_, that was discussed above, may only be effective at low levels of task demands. With the increasing task load required for the DT, cortical processing capacity may have become insufficient, as suggested by the compensation-related utilization of neural circuits hypothesis (CRUNCH) in the aging literature (Reuter-lorenz and Cappell, 2008). This hypothesis assumes a non-linear relationship between brain activity and cognitive load. In line with this model, OA may generate more brain activity at low or intermediate task load (ST) in contrast to high task load (DT) when processing capacity may be exceeded. As a result, OA may generate less neural activity than YA in the high load conditions, which could explain the present findings. Indeed, St George et al. (2021) also showed recently that the change in PFC-HbO_2_ levels from ST to DT were task-load dependent in OA, as HbO_2_ levels increased with easy tasks and decreased with more difficult ones (St George et al., 2021). Finally, the DT-related deactivation could also signify a spread to other (sub)cortical brain areas not captured with the fNIRS setup, such as the cerebellum (Leone et al., 2017).

Figure 6A also highlights that higher HbO_2_ levels were present in the right SMA and SSC (blue regions) in OA compared to YA during the wasp game and irrespective of task load. More online motor planning, right-left coordination, and sensorimotor integration were probably needed with age, due to the loss of motor automaticity (Boisgontier et al., 2013) and neural efficiency (Daselaar et al., 2015) inherent to aging, especially when performing a challenging balance game. This was also reflected in the balance and cognitive test outcomes, as OA had more compromised balance and executive dysfunction than YA.

HHb changes revealed that YA showed more deactivation (less activation) of the left SMA during DT compared to ST_wg_, yet with only low impact on weight-shifting performance. OA, on the other hand, showed less deactivation (more activation) of the left SMA during DT compared to YA, while the %DT-cost on the game performance was significantly higher. Possibly, OA attempted to recruit their SMA to be able to combine weight-shifting with serial subtraction but were unsuccessful because SMA-levels had reached a plateau, as was previously projected by the CRUNCH hypothesis. In contrast to our findings, Stuart et al. (2019a) found that YA demonstrated higher SMA activity during DT-gait than OA, which was ascribed to a stronger reliance on the communication between the SMA and the basal ganglia (Redgrave et al., 2010) in YA. In the present paradigm, the increased SMA activity in OA could point to a greater reliance on this cortical region when engaged in the wasp game, as it involves a more goal-directed action than gait. Unfortunately, the fNIRS system does not allow capturing subcortical activity, and therefore making inferences about subcortical involvement is not possible.

#### Summed ST vs. DT Cortical Activations

Figure 6B illustrates that when HbO_2_ levels during DT were compared to ST_wg_+ST_ss_, global reductions in most ROIs (orange regions) were found except for the right PFC and this regardless of age. This indicates that the DT-processing tended to deactivate the regions under investigation in both age groups. In a systematic review of whole brain fMRI data, Leone et al. (2017) compared DT vs. summed ST-contrasts and found highly inconsistent results. Most studies revealed an over-additive activation pattern, revealing extra recruitment needed for DT in specific brain areas responsible for task integration, such as the cerebellum. However, another group of studies demonstrated relative under-additive activation during DT-processing, which was taken to mean that resource competition was at play especially when they concurred with DT deterioration. To date, only one study applied this analysis to fNIRS in selected ROIs during dynamic stance combined with an auditory choice reaction time task. In analogy to the present study, DT-related reductions of HbO_2_ levels were found in all ROIs when compared with the summed ST-activations (Rosso et al., 2017). Partially similar to the present results, Rosso et al. (2017) demonstrated that this pattern of deactivation was apparent in both YA and OA. However, the present study found greater under-additive activation in OA than in YA in the left PMC and FEF and the right SMA (green regions) in conjunction with reduced proficiency in the DT task performance. In addition, Pelicioni et al. (2019) found a reduction in cortical activity when the complexity of a stepping task increased, accompanied by a slowing down in performance (Pelicioni et al., 2020). These results may reflect that OA displayed enhanced neural competition (Roland and Zilles, 1998) in these regions, which contributed to the DT-deficits.

HHb levels in the right FEF mirrored this pattern of relative decline of neural flexibility, as YA deactivated this area more during the DT in contrast to OA. Altogether, this reduced availability of neural resources in motor areas as well as visual attention regions may explain why OA were slow in weight-shifting, hit less wasps and had worse serial subtractions scores, albeit that correlational analysis did not confirm a direct link with behavior.

### Limitations and Strengths

Some limitations have to be taken into consideration when interpreting the present results. Sample size was determined pragmatically and was not power-based, which could have influenced the results' estimates. We determined the functional limits of stability for calibration of the wasp game before putting on the fNIRS cap. As the weight of the fNIRS cap and cables was not taken into account, this may have had an impact on weight-shifting performance. However, it is unlikely that it affected performance between tasks and groups differently. For this analysis, we adhered to recently published recommendations (Vitorio et al., 2017; Menant et al., 2020) and also conducted a complementary HbO_2_-HHb-correlation analysis as well as a reliability check of visual inspection of the fNIRS signals between testers. Both blood volume and blood flow can influence the HbO_2_-HHb-correlation, the overall value of which is likely to be negative (Scholkmann et al., 2014). Posture can cause changes in blood pressure (Tachtsidis et al., 2004). This factor could not have influenced our results, as the block frequency was lower than the typical Mayer wave frequency of 0.1 Hz (Yücel et al., 2016). As well, no difference across conditions between fNIRS outcomes were likely as the active task was compared to a baseline task and both were carried out in the upright position. Furthermore, when converting optical density to HbO_2_ and HHb using Beer-Lamberts law, the differential path length factor is shown to be dependent on age. As this factor was currently unknown for OA aged above 50 years, the same factor was used for this group throughout the experiment. Another limitation was that we did not take the participants' physical activity levels into account, although it has been demonstrated to influence fNIRS responses in both YA and OA (Dupuy et al., 2015). Although our fNIRS setup did not include short-separation channels to correct for extra-cerebral components, we performed an anti-correlation analysis and applied recommended filters to improve signal quality (Jang et al., 2009). Finally, no fMRI scans of the participants were available. However, using a 3D-digitizer in combination with the spatial registration routine in NIRS-SPM software, we were able to identify multiple channels for each ROI.

### Clinical Implications

The results of this study point to several clinical implications. The findings suggest that high levels of task complexity may be contra-indicated in OA due to the risk of adversely impacting interactions between cognitive and motor neural networks. This should be taken into account when using popular game-like training environments. However, cognitive distraction and dual-tasking is highly relevant for daily life activities. Therefore, future studies need to investigate whether balance games with or without a DT can enhance neural flexibility in OA and whether this leads to better balance and reduced falls in daily life.

## Conclusion

This study provides new insights into age-related cortical activation patterns during a game-like postural control task with and without serial subtraction. Despite adjustments to each individual's limits of stability, game performance as well as serial subtraction scores deteriorated with age when performing both tasks simultaneously. Moreover, visual, cognitive and motor cortical regions were recruited differently during DT with age, suggesting that competitive neural deactivation contributed to the DT-interference.

## Data Availability Statement

The datasets generated for this article are not readily available because of ethical and privacy reasons. Requests to access the datasets should be directed to veerle.derond@kuleuven.be.

## Ethics Statement

The studies involving human participants were reviewed and approved by Newcastle University Ethics Committee. The patients/participants provided their written informed consent to participate in this study.

## Author Contributions

VdR was responsible for the protocol, recruitment, data collection, data analysis, and writing the manuscript. DO-S contributed to data collection and data analysis. BD helped with setting up the protocol and provided advice throughout the study. AN supervised and J-JO co-supervised the study as part of a Ph.D. trajectory at KU Leuven. AP supervised the experimental set-up and data collections at Newcastle University. All authors approved the final version of this manuscript.

## Conflict of Interest

The authors declare that the research was conducted in the absence of any commercial or financial relationships that could be construed as a potential conflict of interest.

## Abbreviations

YA: young adults
OA: older adults
ST: single-task
ST_wg_: single-task wasp game
ST_ss_: single-task serial subtractions
DT: dual-task
fNIRS: functional Near-Infrared Spectroscopy
ROI: region of interest
HbO_2_: oxygenated hemoglobin
HHb: deoxygenated hemoglobin
PFC: pre-frontal cortex
FEF: frontal eye fields
PMC: premotor cortex
SMA: supplementary motor area
SSC: somatosensory cortex
CoM: center of mass
ML: mediolateral.
