# Supplementary material for: Compromised Brain Activity With Age During a Game-Like Dynamic Balance Task: Single- vs. Dual-Task Performance
Source: Front Aging Neurosci. 2021 Jul 5;13:657308. doi: 10.3389/fnagi.2021.657308 (PMC8287632; doi:10.3389/fnagi.2021.657308)
Supplement: Supplementary file 1 [file Data_Sheet_1.PDF]

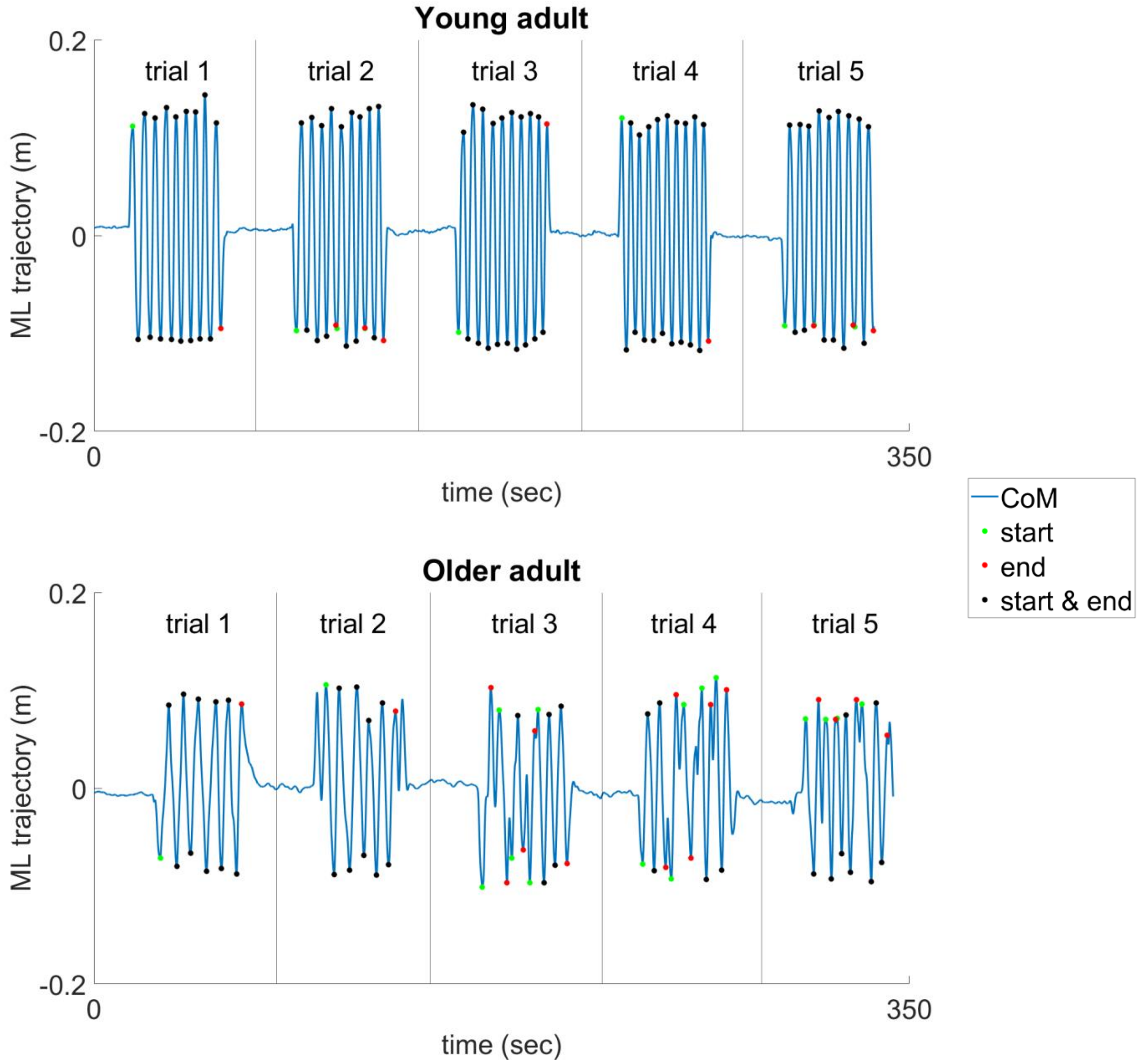

Supplementary Figure 1. Weight-shifting trajectory during one ST block for a representative young and older adult in the ML direction. Green stars indicate the start and red stars the end of each weight-shift. The black stars indicate that the start at the end of a weight-shift was the same. The blue line represents the CoM trajectory. Weight-shifts to the right are displayed as positive and to the left as negative values. ST = single-task; ML = mediolateral; CoM = center of mass.

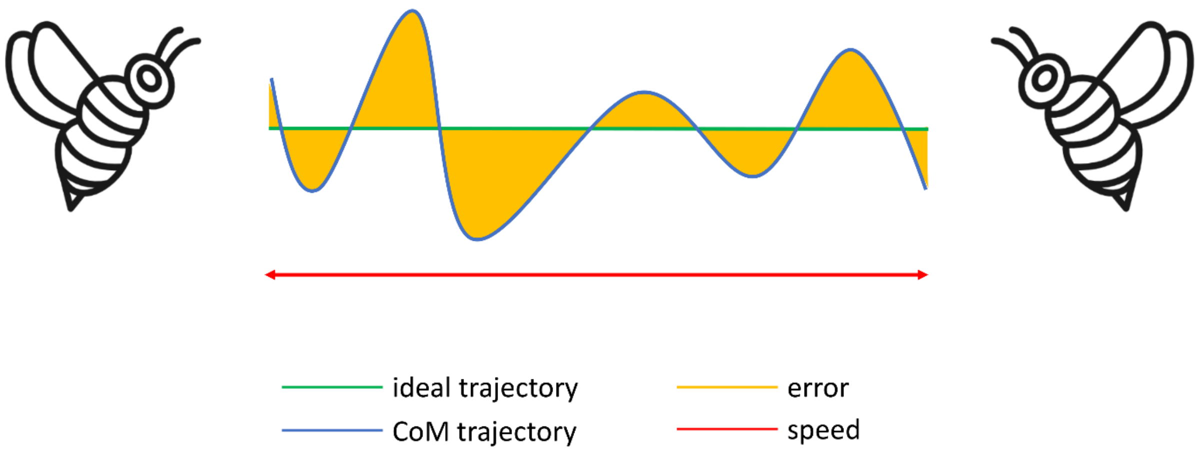

Supplementary Figure 2. Visualization of CoM outcome parameters. The blue line represents the CoM weight-shifting trajectory. The yellow surfaces indicate the weight-shifting error, defined as the deviation in the anterior-posterior direction between the CoM trajectory and the ideal trajectory (i.e. green line). The red horizontal arrow refers to weight-shifting speed in the mediolateral direction. CoM = center of mass.

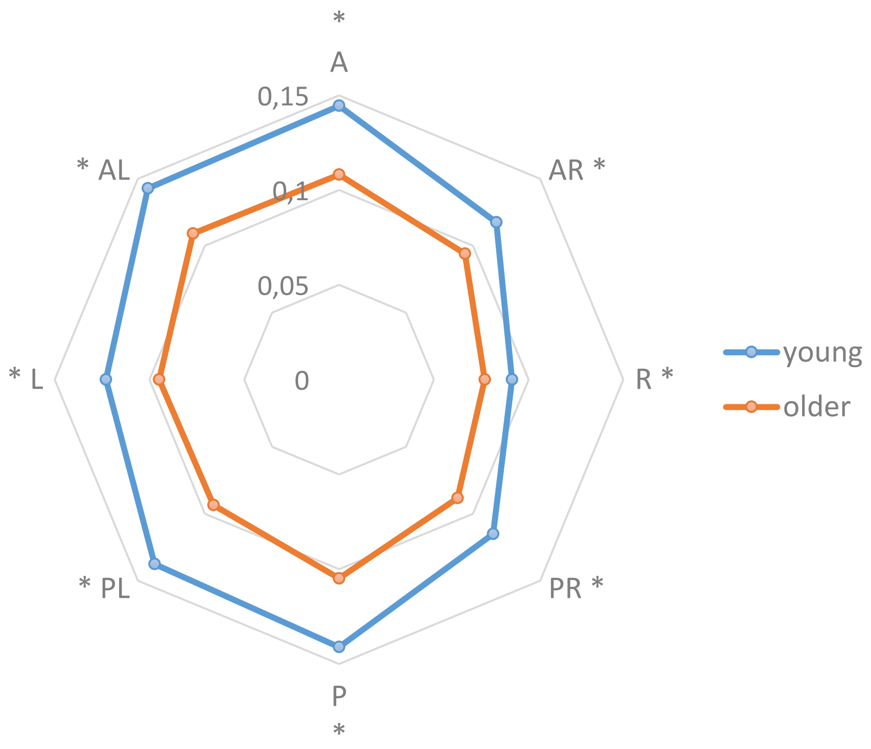

Supplementary Figure 3. Functional limits of stability in eight directions for young and older adults. The blue line represents the average functional limits of stability of young adults and the orange line reflects those of older adults. The eight corners of the spider plot represent the excursions in meters in eight directions: anterior (A), anterior-right (AR), right (R), posterior-right (PR), posterior (P), posterior-left (PL), left (L), and anterior-left (A). *significant age differences (p<.01).

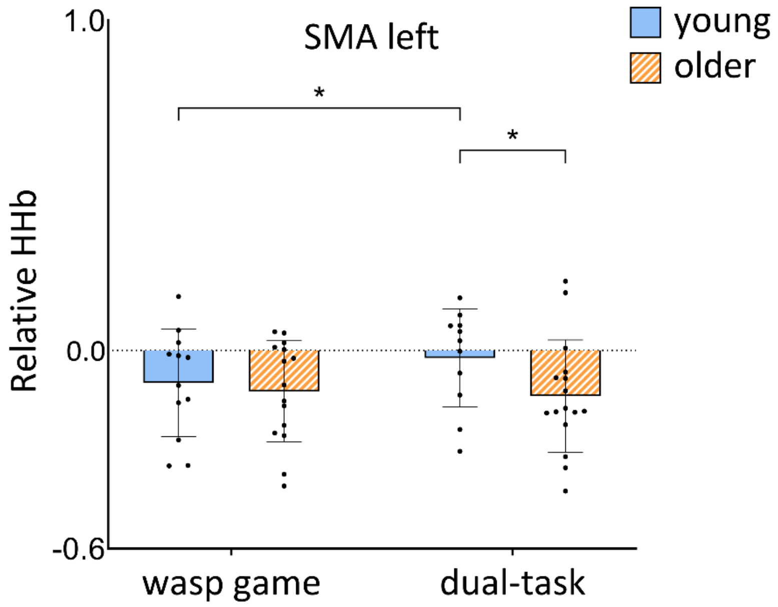

Supplementary Figure 4. SMA left HHb levels for young and older adults during wasp game ST and DT. HHb levels are calculated relative to the baseline task and displayed as mean ± SD. Individual data points represent relative HHb levels for each participant. *Significant post-hoc effects ($p<.05$). SMA = supplementary motor area; HHb = deoxygenated hemoglobin; ST = single-task; DT = dual-task.

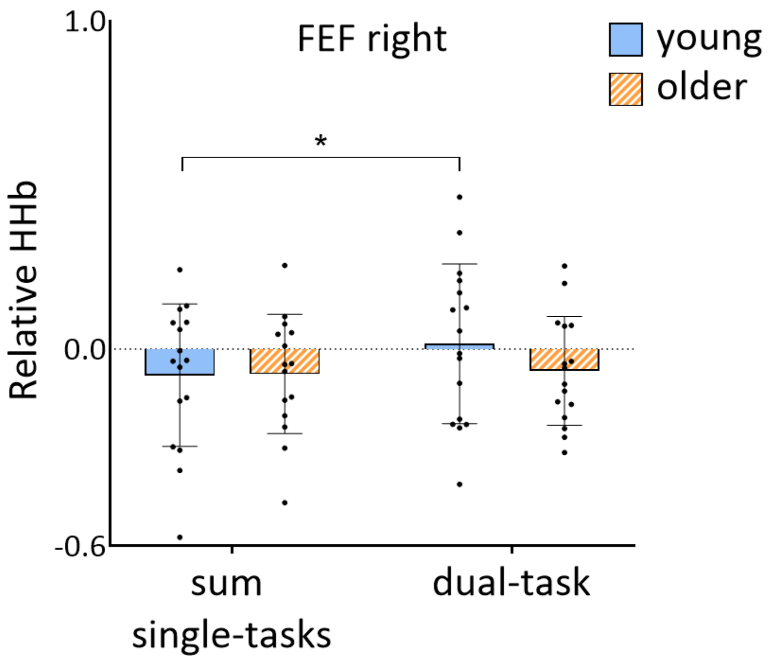

Supplementary Figure 5. FEF right HHb levels for young and older adults in the sum of STs and DT. HHb levels are calculated relative to the baseline task and displayed as mean ± SD. Individual data points represent relative HHb levels for each participant. *Significant post-hoc effects ($p<.05$). FEF = frontal eye fields; HHb = deoxygenated hemoglobin; ST = single-task; DT = dual-task.

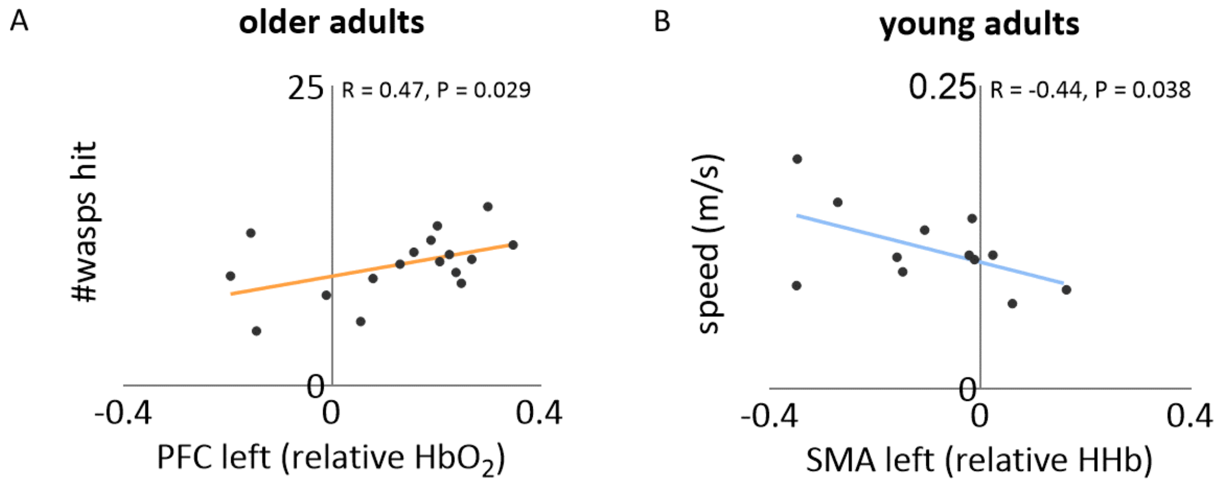

Supplementary Figure 6. Correlation between the number of wasps hit and relative HbO_2 levels in PFC left for older adults **(A)**, and weight-shifting speed and relative HHb levels in SMA left for young adults **(B)** during ST. Both HbO_2 and HHb levels are calculated relative to the baseline task. PFC = prefrontal cortex; SMA = supplementary motor area; ST = single-task; HbO_2 = oxygenated hemoglobin; HHb = deoxygenated hemoglobin.

Supplementary Table 1. Relative HbO_2 levels during wasp game ST and DT for young and older adults

	Young adults[a]		Older adults[a]		Group	Task	Interaction
	**ST**	**DT**	**ST**	**DT**	**main effect**	**main effect**	**effect**
PFC							
left	**-0.033 ± 0.229***	-0.003 ± 0.221	**0.126 ± 0.230*†**	**0.036 ± 0.197†**	**0.029**	**0.035**	**0.003**
right	-0.023 ± 0.240	-0.023 ± 0.279	0.129 ± 0.218	0.097 ± 0.171	0.154	0.408	0.278
FEF							
left	0.071 ± 0.253	0.129 ± 0.288	**0.135 ± 0.213†**	**0.083 ± 0.190†**	0.587	0.529	**0.022**
right	0.021 ± 0.283	-0.022 ± 0.288	0.129 ± 0.229	0.145 ± 0.196	0.068	0.538	0.165
PMC							
left	0.095 ± 0.271	0.093 ± 0.244	0.257 ± 0.195	0.190 ± 0.190	0.132	0.653	0.161
right	0.139 ± 0.264	0.109 ± 0.268	0.162 ± 0.195	0.105 ± 0.212	0.615	0.391	0.350
SMA							
left	0.130 ± 0.210	0.177 ± 0.256	0.174 ± 0.179	0.192 ± 0.194	0.346	0.088	0.456
right	-0.060 ± 0.238	-0.021 ± 0.266	0.187 ± 0.204	0.184 ± 0.161	**0.001**	0.465	0.279
SSC							
left	0.025 ± 0.227	0.025 ± 0.194	0.123 ± 0.226	0.092 ± 0.207	0.054	0.588	0.622
right	-0.012 ± 0.233	-0.028 ± 0.180	0.169 ± 0.181	0.140 ± 0.154	**0.002**	0.053	0.918

Note: Negative values represent a reduction and positive values represent an increase in HbO_2 compared to the baseline task. Values are displayed as mean ± SD. ST = single-task; DT = dual-task. [a]N = 17.
Bold values indicate significant group main, task main and group*task interaction effects (p<.05). *Significant post-hoc group-effect in ST and/or DT. †Significant post-hoc task-effect in young and/or older adults.

Supplementary Table 2. Relative HHb levels during wasp game ST and DT for young and older adults

	**Young adults[a]**		**Older adults[a]**		**Group**	**Task**	**Interaction**
	**ST**	**DT**	**ST**	**DT**	**main effect**	**main effect**	**effect**
PFC							
left	**0.059 ± 0.199***	-0.003 ± 0.248	**-0.069 ± 0.232*†**	**0.016 ± 0.198†**	0.122	0.197	**0.001**
right	-0.018 ± 0.256	-0.043 ± 0.260	-0.142 ± 0.194	-0.116 ± 0.154	0.342	0.971	0.126
FEF							
left	-0.036 ± 0.267	-0.035 ± 0.329	-0.118 ± 0.179	-0.092 ± 0.203	0.167	0.390	0.782
right	**-0.045 ± 0.253†**	**0.019 ± 0.287†**	-0.047 ± 0.199	-0.070 ± 0.205	0.460	0.500	**0.041**
PMC							
left	0.018 ± 0.220	-0.031 ± 0.246	-0.141 ± 0.186	-0.082 ± 0.232	**0.030**	0.808	0.072
right	-0.139 ± 0.271	-0.130 ± 0.269	-0.106 ± 0.176	-0.046 ± 0.197	0.287	0.408	0.228
SMA							
left	**-0.098 ± 0.213†**	**-0.022 ± 0.212*†**	-0.124 ± 0.191	**-0.138 ± 0.213***	**0.020**	0.091	**0.018**
right	0.132 ± 0.263	0.099 ± 0.277	-0.076 ± 0.222	-0.093 ± 0.175	**<0.001**	0.948	0.752
SSC							
left	0.086 ± 0.267	0.022 ± 0.229	-0.031 ± 0.196	-0.030 ± 0.157	0.058	0.103	0.257
right	0.002 ± 0.159	0.086 ± 0.238	-0.051 ± 0.224	-0.016 ± 0.206	0.167	0.525	0.499

Note: Negative values represent a reduction and positive values represent an increase in HHb compared to the baseline task. Values are displayed as mean ± SD. ST = single-task; DT = dual-task. [a]N = 17.
Bold values indicate significant group main, task main and group*task interaction effects (p<.05). *Significant post-hoc group-effect in ST and/or DT. †Significant post-hoc task-effect in young and/or older adults.

Supplementary Table 3. Relative HbO_2 levels of the sum of STs and DT for young and older adults

	**Young adults[a]**		**Older adults[a]**		**Group**	**Task**	**Interaction**
	**ST+ST**	**DT**	**ST+ST**	**DT**	**main effect**	**main effect**	**effect**
PFC							
left	-0.011 ± 0.303	-0.003 ± 0.221	0.131 ± 0.272	0.036 ± 0.197	**0.030**	**0.035**	0.053
right	-0.027 ± 0.326	-0.023 ± 0.279	0.150 ± 0.307	0.097 ± 0.171	0.058	0.352	0.199
FEF							
left	0.109 ± 0.293	0.129 ± 0.288	**0.255 ± 0.303**†	**0.083 ± 0.190**†	0.436	**<0.001**	**<0.001**
right	0.099 ± 0.309	-0.022 ± 0.288	0.190 ± 0.320	0.145 ± 0.196	0.066	**<0.001**	0.112
PMC							
left	**0.096 ± 0.350***	0.093 ± 0.244	**0.379 ± 0.279***†	**0.190 ± 0.190**†	0.073	**0.018**	**0.002**
right	0.166 ± 0.344	0.109 ± 0.268	0.208 ± 0.300	0.105 ± 0.212	0.614	**0.043**	0.178
SMA							
left	0.212 ± 0.282	0.177 ± 0.256	0.285 ± 0.265	0.192 ± 0.194	0.441	**0.012**	0.070
right	**-0.020 ± 0.295***	**-0.021 ± 0.266***	**0.281 ± 0.280***†	**0.184 ± 0.161***†	**0.001**	**0.024**	**0.020**
SSC							
left	0.077 ± 0.325	0.025 ± 0.194	0.220 ± 0.276	0.092 ± 0.207	**0.038**	**<0.001**	0.593
right	0.060 ± 0.295	-0.028 ± 0.180	0.268 ± 0.215	0.140 ± 0.154	**0.005**	**<0.001**	0.198

Note: Negative values represent a reduction and positive values represent an increase in HbO_2 compared to the baseline task. Values are displayed as mean ± SD. ST = single-task; DT = dual-task. [a]N = 17.
Bold values indicate significant group main, task main and group*task interaction effects (p<.05). *Significant post-hoc group-effect in ST and/or DT. †Significant post-hoc task-effect in young and/or older adults.

Supplementary Table 4. Relative HHb levels of the sum of STs and DT for young and older adults

	**Young adults[a]**		**Older adults[a]**		**Group**	**Task**	**Interaction**
	**ST+ST**	**DT**	**ST+ST**	**DT**	**main effect**	**main effect**	**effect**
PFC							
left	0.046 ± 0.262	-0.003 ± 0.248	-0.045 ± 0.306	0.016 ± 0.198	0.118	0.654	0.084
right	-0.003 ± 0.337	-0.043 ± 0.260	-0.133 ± 0.302	-0.116 ± 0.154	0.137	0.658	0.298
FEF							
left	-0.048 ± 0.301	-0.035 ± 0.329	-0.155 ± 0.283	-0.092 ± 0.203	0.270	**0.036**	0.225
right	**-0.080 ± 0.294**†	**0.019 ± 0.287**†	-0.076 ± 0.274	-0.070 ± 0.205	0.693	0.122	**0.022**
PMC							
left	0.049 ± 0.268	-0.031 ± 0.246	-0.129 ± 0.325	-0.082 ± 0.232	0.067	0.405	0.087
right	-0.170 ± 0.295	-0.130 ± 0.269	-0.067 ± 0.257	-0.046 ± 0.197	0.052	0.697	0.985
SMA							
left	-0.093 ± 0.260	-0.022 ± 0.212	-0.125 ± 0.304	-0.138 ± 0.213	0.107	0.306	0.399
right	0.0912 ± 0.321	0.099 ± 0.277	-0.052 ± 0.268	-0.093 ± 0.175	**0.001**	0.886	0.395
SSC							
left	0.0360 ± 0.312	0.022 ± 0.229	-0.038 ± 0.264	-0.030 ± 0.157	0.104	0.573	0.733
right	-0.029 ± 0.265	0.086 ± 0.238	-0.040 ± 0.294	-0.016 ± 0.206	0.277	0.320	0.381

Note Negative values represent a reduction and positive values represent an increase in HHb compared to the baseline task. Values are displayed as mean ± SD. ST = single-task; DT = dual-task. [a]N = 17.
Bold values indicate significant group main, task main and group*task interaction effects (p<.05). *Significant post-hoc group-effect in ST and/or DT. †Significant post-hoc task-effect in young and/or older adults.
